# The role of grievance in fatal family violence and implications for the construct of lone actor grievance-fuelled violence

**DOI:** 10.3389/fpsyg.2022.1057719

**Published:** 2022-12-15

**Authors:** Alannah J. Cooper, Michele T. Pathé, Troy E. McEwan

**Affiliations:** ^1^Centre for Forensic Behavioural Science, Swinburne University of Technology and Forensicare, Alphington, VIC, Australia; ^2^Centre of Research and Education in Forensic Psychology, University of Kent, Canterbury, United Kingdom

**Keywords:** grievance-fuelled violence, fatal family violence, lone actor, family violence, grievance, intimate partner homicide, filicide, femicide

## Abstract

**Introduction:**

The concept of lone actor grievance fuelled violence assumes that homicides that occur in very different contexts can be thought about in a consistent manner because they share common motivations and resultant emotional states like resentment, outrage or revenge. Fatal family violence has been largely excluded from discussions of lone actor grievance-fuelled homicide, based on the assumption that it is conceptually different. This scoping review examines similarities and discrepancies between the characteristics and motivations of perpetrators of fatal family violence and those who have engaged in lone actor grievance-fuelled homicide outside the family context, and the relevance of the concept of grievance-fuelled violence to fatal family violence.

**Methods:**

This study reviewed published case studies and case series, resulting in a dataset of 102 homicide cases from 36 studies, of which there were 38 fatal family violence cases and 64 categorised as lone actor grievance-fuelled homicide.

**Results:**

Twenty of the 38 fatal family violence cases were identified as being grievance-fuelled, based on the presence of motivations consistent with definitions in the grievance literature. Whilst there were some offence similarities between the fatal family violence cases (e.g., location of offence), those driven by grievance were more similar to lone actor grievance-fuelled homicide in other ways (e.g., offender’s gender and offence methods). In both these categories violence was predominantly motivated by grievance and a desire for revenge, whereas non-grievance fatal family violence cases were predominantly motivated by altruism.

**Discussion:**

The motivations that defined behaviour as lone actor grievance-fuelled homicide were equally apparent in a sub-group of fatal family violence, implying that some family violence cases can be integrated into the construct of lone actor grievance-fuelled homicide in future research and theorising.

## Introduction

On 13 August 2021, Jake Davison, 22, fatally shoot his mother in their home in Plymouth, southern England, before stepping out onto the street and shooting dead a man and his three-year-old daughter who were walking their dog. He then shot and killed a man and woman at a nearby sportsground, before turning the gun on himself. Davison was a self-proclaimed “Incel” (involuntary celibate), an online subculture based on extreme patriarchal and misogynistic views. He had a known history of conflict with his mother, and his online posts depicted his sexual frustration, especially his inability to find a girlfriend, and his hatred of single mothers, including his own (Duell, 2021; Ross, 2021). The co-occurrence of fatal family violence and a public mass killing observed in this case has been previously documented, although it has received little attention to date in the literature. Other contemporary cases with similar characteristics include Nikolas Cruz, who had a well-documented history of abusing his mother and ex-girlfriend. He had expressed pro-Incel views prior to killing his ex-girlfriend and 17 other people at his former high school in Florida on Valentine’s Day, 2018 (Issa, 2019; Marganski, 2019; Hoffman et al., 2020). Similarly, when the aggrieved and later ISIS-inspired Man Haron Monis seized 18 hostages in Sydney’s Lindt Café siege in December 2014, he was on bail for orchestrating the murder of his second wife, had a documented history of abusing his first wife, and was facing charges for 40 sexual offences involving other women (Coroners Court of New South Wales [NSW], 2017). There are numerous other cases described in the literature in which grievance-fuelled violence by lone individuals was preceded or accompanied by family and/or intimate partner violence (Guzman-Lopez, 2011; Issa, 2019; Smith, 2019).

Lone actor grievance-fuelled violence (LAGFV) is an overarching term that includes “individuals who commit violent acts motivated by idiosyncratic grievances, underpinned by a sense of injustice, loss, injury, or victimisation” (Pathé et al., 2018, pp. 38–39). The current study focuses specifically on acts of LAGFV that resulted in *at least one fatality*, excluding the perpetrator, and so refers to lone actor grievance-fuelled *homicide* (LAGFH) throughout. Several forms of LAGFV/H have been described in the literature, including lone actor terrorist acts, hate killings, workplace and school killings, and public figure assassinations (Pathé et al., 2018).

The concept of LAGFV/H has attracted increasing interest over the past decade (Barry-Walsh et al., 2020; Ebbrecht, 2022). As in Davison’s case, there is evidence that family members may be targeted as part of a wider act of LAGFH, and that some cases have a prior history of family violence (De Voe and Nicholson, 2020; Everytown for Gun Safety, 2021). To date, there has been a tendency to regard violence and homicide within the family and LAGFH which targets individuals outside the family as separate domains (McCulloch et al., 2019). Whilst a few studies have included familicides or family massacres in discussions of LAGFH (McCauley et al., 2013; Capellan, 2015; Hurlow et al., 2016), the literature has tended to treat fatal family violence (FFV; *deliberate homicide of at least one family member, including extended family, guardians, and current or former intimate partners*) as conceptually distinct (Lankford, 2012; Krouse and Richardson, 2015; Clemmow et al., 2020) and these fields of research have developed separately. This is somewhat surprising as there are more than anecdotal grounds to consider that a grievance or multiple grievances may be relevant to some acts of FFV. For example, Harden et al.’s (2019) recent thematic synthesis of 20 studies of intimate partner homicide identified that two common motivations were jealousy and revenge for relationship termination.

### The construct of lone actor grievance-fuelled violence

The descriptors LAGFV and LAGFH are relatively new, though the behaviours they capture have been discussed for many years (Clemmow et al., 2020). The “lone actor” element originated in descriptions of individuals committing acts of terrorism or ideologically-motivated violence outside the context of a terrorist organisation or cell (Corner and Gill, 2015; Corner et al., 2016, 2018). Whilst the definition of a “lone actor” varies (Kenyon et al., 2021), lone actor terrorism can be considered one form of LAGFV, sharing key characteristics with individuals who engage in apolitical attacks fuelled by personal grievances (Clemmow et al., 2020). The significance of grievances to lone actor violence was originally discussed in studies of individuals who threatened, stalked and attacked public figures, many of whom were motivated by a highly personal cause or grievance (James et al., 2009; Mullen et al., 2009).

Research into lethal and near lethal mass violence throughout the 2010s increasingly found that lone perpetrators of extreme violence shared some key similarities both within similar (see Ioannou et al., 2015) and different contexts. Lankford (2012) was one of the earliest to compare lone actor terrorists with those engaging in public mass killings at schools or workplaces, concluding that most distinctions between these offenders were superficial and precipitating crises were common across all four groups. McCauley et al. (2013) compared lone actor terrorists to public figure assassins and school shooters, suggesting that the common theme of a perceived grievance – as opposed to material self-profit – rendered these groups more similar than different. Capellan (2015) subsequently reviewed a sample of US active shooters, finding that ideological and non-ideological active shooters share very similar personal profiles, with differences relating only to the preparation and execution of the attack. Capellan et al. (2019) reviewed mass shooters in different contexts (workplace, school, ideologically-motivated, and public rampage), noting several similarities, as well as differences in relation to specific motivations, demographic factors, and the level of attack planning. Böckler et al. (2018) also acknowledged the many similarities between lone actor terrorists and school shooters, including escalation patterns and the execution of the attack. This was further observed by Clemmow et al. (2020) in their review of lone actor terrorists and mass murderers. They proposed that these crises or other situational considerations motivate the individual towards the grievance narrative, rather than being caused by the extreme ideology itself (p. 15). In their review, Barry-Walsh et al. (2020) observed that individuals whose actions could be conceptualised as LAGFV (school/university killers, workplace, killers and lone-actor terrorists) had overlapping characteristics, such as a grievance propelled by a perception of mistreatment by a person or wider society. Pathé et al. (2018) noted that those with pathological fixations, and apolitical mass killers “typically all harbour some personal grievance or vendetta, triggered by a perceived injustice” (p. 39). The construct of LAGFV has now gained wide acceptance as a useful framework for the systematic study of “acts of demonstrative violence perpetrated by a single offender” (Ebbrecht, 2022).

### Fatal family violence as a form of LAGFV/H

The lack of attention in the reviewed literature to FFV or family violence more broadly is surprising on the basis that, like FFV, LAGFV is highly gendered and involves severe violence. LAGFV is almost entirely the province of male perpetrators and some authors have suggested that gender and gender-related factors should be given a more central role in understanding the mechanisms underpinning LAGFV/H (McCulloch et al., 2019; Scaptura, 2019; DeVoe and Nicholson, 2020; Rottweiler et al., 2021; Silva et al., 2021). McCulloch et al. (2019) undertook a gendered analysis of several cases of lone actor terrorism, noting the lack of attention to gender and violence against women in this field, despite the availability of information. Drawing on a detailed case study of Man Monis, these authors argued that because violence against women is not considered “real violence” its significance has been overlooked in LAGFV research. They noted that this is consistent with the wider criminological research, where domestic violence is seen as a uniquely female phenomenon, different and separate to other forms of violence.

There is reason to think that the two constructs, FFV and LAGFV/H, may substantially overlap. Two studies suggest that a large proportion of those who engage in LAGFV/H have histories of family violence or violence against women. DeVoe and Nicholson (2020) conducted a review of mass shootings in the United States between 1966 and 2020. They suggested that almost half of all public mass shooters had known histories of violence against women, and this figure rose to 90% for the deadliest mass shootings. Others found that 53% of mass shootings in the United States involved the offender shooting an intimate partner or family member in addition to other victims (Everytown for Gun Safety, 2021). Two other reviews suggest that broader gender-related factors are relevant to a substantial sub-group of LAFGH. Silva et al. (2021) specifically examined gender-based mass shootings, finding that 34% of all public mass shootings in the United States between 1966 and 2018 were motivated by grievances against women. When confined to the years 2010 to 2018, that increased to 45%. This statistic is particularly concerning given these researchers excluded killings occurring exclusively in the home and family. Recognising the overlap between violent extremism, violence against women, and family violence, Rottweiler et al. (2021) suggested that even when violence against women is not the sole motive, misogyny is often a factor in grievances that form the basis for wide-ranging attacks.

### The current study

There is increasing anecdotal and some empirical evidence for conceptual similarities between resentment-based forms of family violence and LAGFV/H in a non-familial context. This study reviewed published case studies to address three research questions:

**Research Question 1**: What are the behaviours, characteristics and motivations of those engaging in fatal family violence (FFV) and in lone actor grievance-fuelled homicide (LAGFH), and are they similar or distinct?

**Research Question 2**: What are the defining features that in their presence or absence make an act of FFV grievance-fuelled or not?

**Research Question 3**: How frequently is family violence observed amongst those engaging in LAGFH, or considered relevant to instances of LAGFH?

## Materials and methods

This study examined how LAGFH and FFV are conceptualised and defined in existing research and any co-occurrence of these potentially distinct types of violence. A scoping review was the preferred methodology given the main purpose of the study was to identify a body of available evidence, identify knowledge gaps, and clarify key concepts. The scoping review also enabled mapping of evidence in relation to broader research questions (Munn et al., 2018), rather than focusing on a precise research question from a relatively narrow range of studies (Arksey and O’Malley 2015).

### Definitions

For screening purposes, lone actor grievance-fuelled homicide (LAGFH) was defined as *the deliberate homicide of at least one person by an individual or dyad in a single incident that appeared to be driven by a grievance or sense of injustice, or recorded as a form of lone actor grievance-fuelled violence (LAGFV)*. LAGFV incorporates lone actor terrorism/extremist ideology, lone actor hate, school/university, workplace, and mass public killings. The initial blanket inclusion of these LAGFV “subtypes” in the screening process allowed for further review as to whether cases could be included under assumed grievances. For instance, some cases were deemed grievance-fuelled based on the rationale for very similar offences committed by others. In the example of a school shooter with limited case details other than being previously expelled, a grievance could be deduced in the absence of contradictory information, as this situation was clearly associated with the presence of a grievance in other similar offences.

Acts of fatal family violence (FFV) were defined as *the deliberate homicide of at least one family member, including current or former intimate partners (regardless of marital or relationship status), biological relative (half-sibling, extended family members) or adoptive family, step-relatives, foster parents or guardians*. Where there was evidence that an act of FFV was underpinned by a sense of injustice, loss, injury, or victimisation it was further classified as grievance-fuelled fatal family violence (GF-FFV). In this study FFV without evidence of any grievance was referred to as “non-grievance FFV.”

### Databases and search terms

Studies included in this review were identified using a keyword search of relevant academic electronic databases conducted between 15 and 19 August 2021 (PsychINFO, SCOPUS and PubMed). No date parameters were used to obtain the widest possible scope of articles, and only published articles were reviewed. Books and grey literature were excluded due to accessibility and time limitations in conducting the review.

#### Search terms

The same search terms related to killing (kill* OR homicide OR murder OR shoot* OR massacre OR lethal OR fatal*) AND motivation or personal characteristics of offenders (motiv* OR behav* OR characteristic) were used to conduct searches across seven themes, given the LAFGV/H literature has been published under disparate fields over many years. The seven themes were: terrorism, school, work, hate, mass, family (including intimate partners), and grievance. The full search terms within each theme and filters applied to narrow the search are available from the author on request.

### Screening of studies

**Figure 1:**
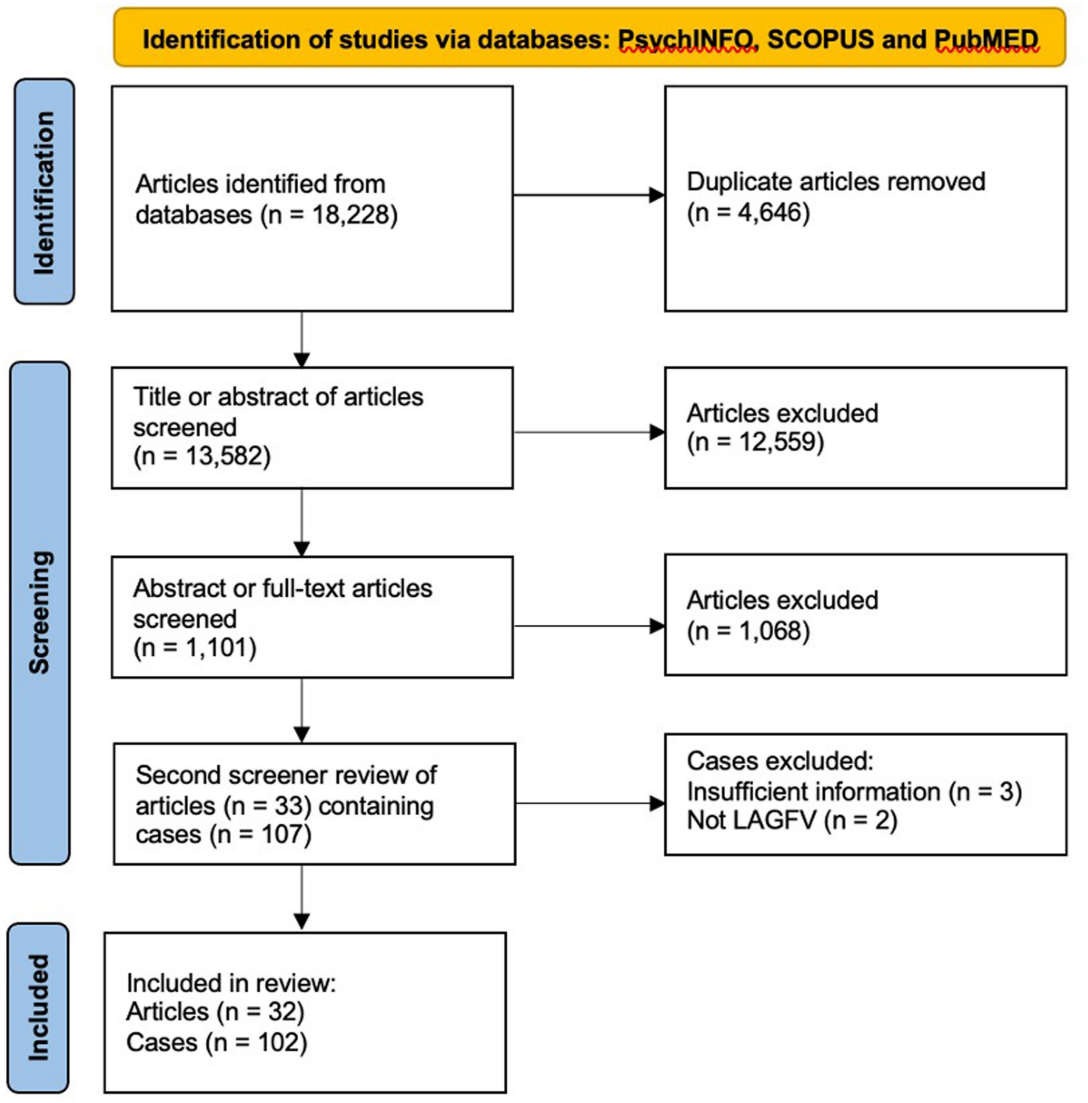
Studies selection flow diagram (PRISMA flow chart).

A PRISMA diagram of study selection is shown in Figure 1. All studies identified in the initial search (*n* = 18,228) were exported into EndNote and the title and abstract (if available *via* EndNote) reviewed for the following inclusion criteria:

- The focus of the study was humans; and
- The study involved the deliberate homicide of at least one family member OR act of LAGFH; and
- The offender acted alone or in an isolated dyad;^1^ and
- Offender characteristics, behaviours and/or motivations were detailed; and
- The paper was available in English, German or Dutch;^2^ and
- The incident occurred in an industrialised, liberal democratic nation.

Book or literature reviews, conceptual papers, population samples, and duplicate studies were excluded. Some high-profile cases were duplicated within the sample and these were not removed as different authors frequently reported different information, enabling more accurate coding. Studies drawn from the sole source of media reporting were excluded unless they provided corroborative evidence that the source was from official records (e.g., court transcripts). All remaining abstracts (i.e., those that had not been available in EndNote) and/or full-text articles (*n* = 1,101) were then reviewed. This screening phase identified some previously overlooked reasons for exclusion, such as attempted homicides, which were frequently included in terrorism studies to maximise sample size. Without the ability to separate attempts from completed homicides, these studies were excluded. Some further studies were also excluded due to insufficient information to assess whether the incident conformed to the LAGFH definition, or if the information was too limited to extract meaningful data. At the end of this phase 33 articles detailing 107 case studies of LAGFH or FFV were included. Articles containing case series were assessed as individual cases and either included or excluded based on available information.

PRISMA flow diagram (Page et al., 2021).

Each case was classified into two mutually exclusive groups as FFV or LAGFH, according to whether the act included the killing of a family member. Forty-one variables were coded in each case, derived from review of LAGFH literature, and the specific aims of the study, and included offence behaviours (e.g., location, weapons, planning), offender characteristics (e.g., gender, violent history, mental health), victim characteristics (e.g., adult or child, gender), and stressors (e.g., relationship, employment). “Free text” sections were used to record initial information about offender motivation (coded independently by AC and TM), then further coded into motivation themes after consultation between the authors. In coding stressors, broad categories were initially created based on themes observed in the existing literature, though stressors were also recorded in free text to ensure comprehensive coding. The free text coding was then converted into themes by the authors. A code book with definitions is available from the authors on request.

Each of the 107 cases was independently reviewed by two authors (AC and MP) to ensure consistency in classifying acts as grievance-fuelled or otherwise. Grievances arising from mental illness were still classified as grievance-fuelled, emphasising that grievance is based on the perceived injustice, illness-based or not. Assumed grievances were observed predominantly within case series, where less information tended to be provided and sometimes no specific motivation was recorded. Because school killings accounted for two thirds of the case series articles, they were over-represented amongst assumed grievances. Grievance was assumed in ten cases of school killings based on the consistent rationale for similar killings in this review. Some school homicides were excluded due to insufficient information to categorise them or where the case did not meet the definition of LAGFH. A final sample of 32 articles were included in the review, containing 102 cases.

Inter-rater reliability (IRR) was assessed using a sample of 21 of the 102 cases (20.6%). Absolute agreement of 93% was reached between one of the authors (AC) and a non-author rater, (agreement in 741 of the 798 coding decisions). Disagreements occurred across 19 variables and were resolved by consultation between raters. Seven variables were discrepant in more than four cases in IRR coding. Review of definitions showed the need for some clarification and these variables were re-coded by the authors for all cases.

## Results

The country in which the incident occurred was available in 99 cases:74 in the United States (74.7%), eight in Germany (8.1%), four in New Zealand (3.9%), three in Canada (2.9%), two each (2%) in Australia, Finland, Italy and Norway, and one case each (1%) in Estonia and Sweden. Date of offence was missing in 16 (15.7%) cases but, where known, ranged between the years of 1865 and 2017. The period between 1997 and 2017 accounted for 69.8% of cases.

Of the 102 cases, 64 (62.7%) were categorised as LAGFH and 38 (37.3%) cases as FFV. The FFV category was further divided into two groups depending on the presence of grievance, yielding 20 fatal cases (52.6%) of family violence that were grievance-fuelled (GF-FFV) and 18 (47.4%) in which there was no evident grievance. Within the GF-FFV category, 6 cases (30%) included the killing of a non-family member. The LAGFH category included 40 “school killers” (62.5%), 11 “terrorism/ideology/hate” (17.2%), 2 “workplace” (3.1%), 6 “other” (9.4%) and 5 “mixed” (7.8%). The mixed category referred to more than one form of LAGFH, such as a workplace and school shooting.

Results within each offender category were grouped into the themes of offence behaviours and victim characteristics.

### Offence behaviours and victim characteristics

Table 1 provides a summary of offence behaviours and victim characteristics.

**Table 1:** Offence behaviours and victim characteristics.

Variable	LAGFH		GF-FFV		Non-grievance FFV	
	*N = 64*		*N = 20*		*N = 18*	
	*N*	*%*	*N*	*%*	*N*	*%*
**Location**						
Outside/Public	7	14.3%	1	5.3%	4	30.8%
Inside (Business or public)	37	75.5%	0	N/A	1	7.7%
Inside (Private residence)	2	4.1%	13	68.4%	8	61.5%
Mix	3	6.1%	5	26.3%	0	N/A
Missing data %	15	23.4%	1	5%	5	27.8%
**Weapon used**						
Yes or possible	58	100%	15	75%	10	71.4%
No	0	N/A	5	25%	4	28.6%
Missing data %	6	9.4%	0	N/A	4	22.2%
**Method of killing**						
Shooting	51	89.5%	7	35%	5	35.7%
Stabbing	3	5.3%	5	25%	2	14.3%
Impact-related injury	3	5.3%	3	15%	4	28.6%
Strangulation or suffocation	0	N/A	4	20%	2	14.3%
Poisoning	0	N/A	1	5%	0	N/A
Drowning	0	N/A	0	N/A	1	7.1%
Missing data %	7	10.9%	0	N/A	4	22.2%
**Offender died at scene**						
Yes, suicide	29	54.7%	3	15.8%	2	12.5%
Yes, other	4	7.5%	0	N/A	0	N/A
No	20	37.7%	16	84.2%	14	87.5%
Missing data %	11	17.2%	1	5%	2	11.1%
**Offence planning**						
Yes or possible	37	100%	11	91.7%	5	55.6%
No	0	N/A	1	8.3%	4	44.4%
Missing data %	27	42.2%	8	40%	9	50%
**Victim type**						
Adult/s	27	69.2%	7	36.8%	6	37.5%
Child/ren	1	2.6%	3	15.8%	9	56.3%
Both	11	28.2%	9	47.4%	1	6.3%
Missing data %	25	39.1%	1	5%	2	11.1%
**Victim gender**						
Female-only	7	38.9%	7	46.7%	8	57.2%
Male-only	7	38.9%	3	20%	3	21.4%
Mix	4	22.2%	5	33.3%	3	21.4%
Missing data %	46	67.6%	5	25%	4	22.2%

#### Location of incident

The major difference between groups was that three quarters of LAGFH incidents occurred inside public or business premises, whilst approximately two-thirds of FFV, grievance-fuelled or otherwise, occurred in private premises.

#### Method of killing

Weapons were used or were likely used to commit homicides in all the LAGFH cases but in fewer (though still the majority) of fatal family violence (GF-FFV 75%; non-grievance FFV 71.4%). Shootings were the main method of killing for LAGFH offenders (89.5%), but were less common in the family context (35% of GF-FFV cases and 35.7% of non-grievance FFV cases). There was a more diverse range of methods used in family killings, including bladed and blunt weapons, strangulation/suffocation, poisoning and drowning.

#### Offence planning and offender death at scene

Offence planning was more frequently reported for the two grievance categories, recorded as present or possibly present in 100% of LAGFH cases and 91.7% of GF-FFV cases, but fewer (55.6%) of non-grievance family killings. There was a large discrepancy in offender death at the scene between the homicide groups, with 62.2% of the LAGFH cohort dying at the scene of the incident – whether by suicide or other means – compared to family homicide offenders (GF-FFV 15.8%; non-grievance FFV 12.5%).

#### Victim characteristics

Information on the age and gender of victims was often lacking, especially as fatality counts increased. Victims were therefore categorised – where known – into adults, children or both. This revealed differences between the three groups, with adults primarily targeted in LAGFH cases (69.2%), adults and children in GF-FFV (47.4%), and mainly children in non-grievance FFV cases (56.3%). In terms of the victim’s gender, female-only victims predominated in family killings, regardless of the presence or absence of grievance (GF-FFV 46.7%; non-grievance FFV 57.2%), whereas no such distinction was seen in the LAGFH cohort (38.9% for both genders, with the remainder missing information).

### Family violence and violent history

Table 2 provides a summary of offender family violence, criminal history and violent behaviour.

**Table 2:** Offender family violence, criminal history and violent behaviours.

Variable	LAGFH		GF-FFV		Non-grievance FFV	
	*N = 64*		*N = 20*		*N = 18*	
	*N*	*%*	*N*	*%*	*N*	*%*
**Non-physical family violence**						
Yes	2	33.3%	1	33.3%	1	50%
Possible^(a)^	4	66.7%	1	33.3%	1	50%
No	0	N/A	1	33.3%	0	N/A
Missing data %	58	90.6%	17	85%	16	88.9%
**Physical family violence**						
Yes	3	60%	2	50%	5	83.3%
Possible^(b)^	2	40%	1	25%	0	N/A
No	0	N/A	1	25%	1	16.7%
Missing data %	59	92.2%	16	80%	12	66.7%
**Family violence-specific criminal charges**						
Yes	2	50%	1	25%	0	N/A
No	2	50%	3	75%	1	100%
Missing data %	60	93.8%	16	80%	17	94.4%
**Family violence or protection order**						
Yes	1	25%	0	N/A	0	N/A
No	3	75%	2	100%	2	100%
Missing data %	60	93.8%	18	90%	16	88.9%
**Criminal record**						
Yes, including violent	3	16.7%	0	N/A	1	50%
Yes, non-violent only	3	16.7%	0	N/A	0	N/A
No	12	66.7%	3	100%	1	50%
Missing data %	46	71.9%	17	85%	16	88.9%
**Unprosecuted problematic behaviours^(c)^**						
Yes, including violent	7	25%	1	25%	0	N/A
Yes, non-violent only	21	75%	3	75%	4	80%
No	0	N/A	0	N/A	1	20%
Missing data %	36	56.3%	16	80%	13	72.2%

^(a)^ Due to the often private nature of family violence offending, some cases provided a clear indication of presence (e.g., direct observations), whilst others were less evident (e.g., opinions from neighbours). ^(b)^ As above. ^(c)^ Behaviours that could result in criminal charges if reported (or other reprimands, such as military punishments). Violent behaviours refer to any form of direct physical assault from the offender to another person. Violence directed at property (e.g., breaking a window) would fall under non-violent.

#### Family violence history

Prior family violence was coded into four categories: non-physical family violence, physical family violence, family violence-specific criminal charges, or family violence protection orders. Only 19 of 102 cases provided information on at least one of these four family violence categories. Similarly, there was limited information in relation to unprosecuted non-physical or physical family violence (16 of 102 cases). Where family violence was mentioned, it tended to be present, but it was not possible to exclude family violence in the absence of information. Given these low numbers, there were no discernible differences between the three offender groups. Most cases also lacked data on family violence-specific criminal charges and protection orders, preventing group comparisons. Of the 19 cases where family violence information was available, a history of family violence was present in 14 (73.7%), whether or not this was formally recorded.

#### Criminal history and unprosecuted problematic behaviours

Twenty-three cases discussed criminal history and 28 unprosecuted violence. Of the 18 cases in the LAGFH cohort that provided this information, 33.4% had a criminal history, with half involving violent offences. Unprosecuted violent behaviours were recorded in a further 25% of these LAGFH cases. Within the family cohorts, none of the GF-FFV offenders were reported to have a criminal record, though this was explicitly discussed in only three cases. In four cases there was a suggestion of unprosecuted criminal acts, both violent and non-violent. Criminal record data was available in only two of the non-grievance FFV cases, though unprosecuted criminal behaviour was reported in four of five cases where it was mentioned. One offender in this group was suspected of a previous homicide, though no arrest or other action was taken.

### Offender characteristics and demographics

#### Gender of offender and country of birth and incident

The offender’s gender was the only variable that was available in all 102 cases. The majority of perpetrators in the grievance categories were male, making up 96.9% of LAGFH and 85% of GF-FFV cohorts. There was a higher proportion of female offenders (55.6%) in the non-grievance FFV category. In 85.7% of cases in both the LAGFH and GF-FFV categories, the offender was born in the same country as the incident, but this was less often the case for offenders in the non-grievance FFV category (62.5%).

#### Offender age and race

Most offenders in the LAGFH category were in the 18–24 year age range (40.4%), whilst offenders in the GF-FFV cohort tended to be younger (31.6% in the 10–17 age range, all school shooters who also murdered family members). Those in the non-grievance FFV category were slightly older, with 38.9% in the 25–34 age range. As shown in Table 3, the majority of offenders were white (66.7% of the LAGFH cohort, 57.1% of the GF-FFV and 55.6% of the non-grievance FFV categories).

**Table 3:** Offender characteristics and demographics.

Variable	LAGFH		GF-FFV		Non-grievance FFV	
	*N = 64*		*N = 20*		*N = 18*	
	N	*%*	N	%	N	%
**Offender gender**						
Male	62	96.9%	17	85%	8	44.4%
Female	2	3.1%	3	15%	10	55.6%
**Offender born in country of incident**						
Yes	12	85.7%	7	87.5%	5	62.5%
Missing data	50	78.1%	12	60%	10	55.6%
**Age**						
10–17	13	27.7%	6	31.6%	2	11.1%
18–24	19	40.4%	5	26.3%	4	22.2%
25–34	6	12.8%	4	21.1%	7	38.9%
35–49	6	12.8%	3	15.8%	3	16.7%
50+	3	6.4%	1	5.3%	2	11.1%
Missing data %	17	26.6%	1	5%	0	N/A
**Offender race**						
White	12	66.7%	4	57.1%	5	55.6%
Black	1	5.6%	2	28.6%	1	11.1%
Asian	2	11.1%	1	14.3%	1	11.1%
Middle eastern	3	16.7%	0	N/A	0	N/A
Other	0	N/A	0	N/A	2	22.2%
Missing data %	46	71.9%	13	65%	9	50%
**Employment**						
Student	13	52%	3	25%	2	25%
Employed	8	32%	5	41.7%	2	25%
Unemployed/homemaker	3	12%	3	25%	4	50%
Pensioner	1	4%	1	8.3%	0	N/A
Missing data %	39	60.9%	8	40%	10	55.6%
**Education**						
Primary	0	N/A	1	14.3%	0	N/A
Early secondary	1	6.3%	3	42.9%	1	16.7%
Upper secondary	10	62.5%	2	28.6%	3	50%
Tertiary	5	31.3%	1	14.3%	2	33.3%
Missing data %	48	75%	13	65%	12	66.7%
**Relationship status**						
Single	12	75%	6	46.2%	2	14.3%
Partner	0	N/A	2	15.4%	0	N/A
Married/defacto	2	12.5%	4	30.8%	9	64.3%
Recently separated	2	12.5%	1	7.7%	3	21.4%
Missing data %	48	75%	7	35%	4	22.2%
**Alcohol during offence**						
Yes or possible	1	33.3%	2	40%	1	100%
No	2	66.7%	3	60%	0	N/A
Missing data %	61	95.3%	15	75%	17	94.4%
**History of alcohol abuse**						
Yes or possible	2	100%	4	66.7%	4	100%
No	0	N/A	2	33.3%	0	N/A
Missing data %	62	96.9%	14	70%	14	77.8%
**Drugs during offence**						
Yes or possible	2	50%	3	42.9%	1	100%
No	2	50%	4	57.1%	0	N/A
Missing data %	60	93.8%	13	65%	17	94.4%
**History of drug abuse**						
Yes or possible	5	83.3%	4	66.7%	3	100%
No	1	16.7%	2	33.3%	0	N/A
Missing data %	58	90.6%	14	70%	15	83.3%

#### Offender employment status and education

Just over half (52%) the LAGFH offenders were students, which was not unexpected given the prominence of school killers in this sample and the lower age range of offenders overall. In terms of educational attainment, 62.5% of the LAGFH cohort had completed upper secondary school level, whilst 31.3% were tertiary educated. GF-FFV offenders had the lowest education rates of the three categories, with 52.7% completing early secondary level or lower and none had any tertiary education. Fifty per cent of offenders in the non-grievance FFV category had upper secondary level education and 33.3% had achieved a tertiary level of education. Employed offenders accounted for 41.7% of the GF-FFV offenders, whilst 50% of offenders in the non-grievance FFV category were said to be unemployed or homemakers.

#### Offender relationship status and relationship to victim

At the time of the homicide, the majority (75%) of offenders in the LAGFH category were recorded as single, whilst this was the case for under half (46.2%) in the GF-FFV category. The relationship status of most non-grievance FFV offenders was recorded as married/defacto (64.3%). The offender’s relationship to the victim was partially identified during the screening process, with FFV cases requiring at least one family member to be a victim. These family victims were wide-ranging, from current and former intimate partners, children and grandparents to extended family members. Within the GF-FFV category, there were five cases (25%) involving both the killing of a family member and at least one person external to the family. Victims in the LAGFH category included formal authority figures, strangers, peers/colleagues, or a combination of these.

#### Offender substance use

Alcohol and drug use were coded in separate themes – alcohol or drugs present in the offender’s system at the time of or shortly prior to the incident, and historical abuse/misuse of alcohol or drugs. Overall, there was insufficient data in all categories to draw any conclusions.

### Offender motivations, stressors and mental illness

As shown in Table 4, the largest difference between grievance and non-grievance homicide groups was observed within offender motivations. The overwhelming theme for LAGFH and GF-FFV was Revenge/Wronged (78.1 and 40%, respectively), whilst the GF-FFV category included three cases with additional motivations (e.g., killing intimate partner for revenge and a child for altruistic reasons). Motivational themes of Self-defence (i.e., the perception that violence was required to defend oneself or others against external threats) were also seen in the GF-FFV cohort, which tended to arise from persecutory symptoms of mental illness. Self-defence was observed in a handful of LAGFH cases but in none of the non-grievance FFV. In contrast, those in the non-grievance FFV category were motivated by altruism, frustration or command hallucinations. Only one offender outside the non-grievance FFV group had any of these motivations. There was no overlap between the LAGFH and non-grievance FFV groups in terms of motivation themes.

**Table 4:** Offender motivations.

	LAGFH		GF-FFV		Non-grievance FFV	
	*N = 64*		*N = 20*		*N = 18*	
	*N*	*%*	*N*	*%*	*N*	*%*
Altruism	0	N/A	0	N/A	8	61.5%
Command hallucinations	0	N/A	0	N/A	3	23.1%
Frustration	0	N/A	1	6.7%	2	15.4%
Self defence	3	9.4%	4	26.7%	0	N/A
Revenge/Wronged	25	78.1%	6	40%	0	N/A
Other	3	9.4%	0	N/A	0	N/A
Multiple	1	3.1%	4	26.7%	0	N/A
Missing or insufficient Information	32	50%	5	25%	5	27.8%

#### Stressors

Table 5 details stressors that were evident prior to the incident. Stressors were defined as something that caused strain or tension in the offender’s life, and these were prominent in all cases. Whilst stress was ubiquitous, the source of stress differed somewhat between the FFV and LAGFH cases. Amongst those who killed a family member, conflict in intimate or familial relationships was recorded as the most pronounced stressor, followed by “mental or physical ill-health or being a victim of abuse” and child custody disputes. Mental/physical illness or being the victim of abuse were also present in a third of the LAGFH cases, but stressors in this group were more commonly idiosyncratic, such as a recent arrest or incarceration, conflict with a specific organisation, racial or religious abuse, homelessness, reputational harm, or the threat of deportation. Social isolation was the second commonest stressor in the LAGFH category (38.9%) but was relatively rare in both fatal family violence groups.

**Table 5:** Offender stressors.

	LAGFH		GF-FFV		Non-grievance FFV	
	*N = 64*		*N = 20*		*N = 18*	
	N	%	N	%	N	%
Stressor present						
Yes	26	65%	15	88.2%	14	82.4%
Possible	14	35%	2	11.8%	3	17.6%
No	0	N/A	0	N/A	0	N/A
Missing data %	24	37.5%	3	15%	1	5.6%
	*N = 36* ^(a)^		*N = 17*		*N = 17*	
	N	%	N	%	N	%
Type of stressor present						
Intimate or familial relationships	12	33.3%	13	76.5%	11	64.7%
Custody or children	1	2.8%	3	17.6%	5	29.4%
Financial	3	8.3%	4	23.5%	4	23.5%
Employment	9	25%	3	17.6%	1	5.9%
School	9	25%	3	17.6%	0	N/A
Mental or physical health/ Victim of abuse^(b)^	13	36.1%	8	47.1%	10	58.8%
Isolation	14	38.9%	2	11.8%	0	N/A
Other	16	44.4%	2	11.8%	3	17.6%

^(a)^ Whilst stressors were present in 40 LAGFH cases, in four cases the nature of the stressor was not identified and therefore has not been included in the above. ^(b)^ Offenders being victims of abuse or trauma were often connected with their mental or physical health within articles. As these were often difficult to separate, they were combined within the one category.

#### Mental illness

The presence of mental illness was coded on the basis of diagnoses received prior to the offending incident, given there is considerable discrepancy in post-incident diagnoses. As shown in Table 6, data on prior mental illness was available in 51 cases, and was highly prevalent, accounting for nearly half of the LAGFH category, nearly two-thirds of the GF-FFV category, and three quarters of the non-grievance FFV group. Psychosis and mood disorders were the most common diagnoses, and the LAGFH and FFV cases could be differentiated by a higher prevalence of psychosis in the latter group, typically in cases that did not involve intimate partners as the homicide victim. Conversely, the LAGFH group were more likely to have “other” diagnoses, usually personality disorder. Amongst the 21 cases in which information on mental status was available but there was no diagnosis prior to the incident, approximately half in each homicide group had a suspected diagnosis based on available information or a diagnosis made after the incident. For example, when an offender died at the scene and had no previous mental health contact, some authors reviewed interviews and other sources of information such as diary entries to retrospectively diagnose suspected mental illness. Of the cases listed as “Not diagnosed” prior to the incident, 50% of the LAGFH and non-grievance FFV categories had a suspected mental illness, as did 40% of GF-FFV cases. Taking these figures and missing data into consideration, the base rate of diagnosed mental disorder was 47.8% in the LAGFH group, 61.5% in GF-FFV, and 73.3% in the non-grievance FFV group.

**Table 6:** Offender mental illness diagnoses preceding homicide.

Variable	LAGFH		GF-FFV		Non-grievance FFV	
	*N = 64*		*N = 20*		*N = 18*	
	N	%	N	%	N	%
Diagnosed	11	47.8%	8	61.5%	11	73.3%
Psychotic disorder	0	NA	5	62.5%	5	45.5%
Mood disorder	2	33.3%	2	25%	5	45.5%
Other/Not stated	4	66.7%	1	12.5%	1	9.1%
Not diagnosed^(a)^	12	18.8%	5	38.5%	4	26.7%
Suspected mental illness^(b)^	6	50%	2	40%	2	50%
Missing data %	41	64.1%	7	35%	3	16.7%

^(a)^ Refers to the absence of a known mental illness diagnoses present prior to or present during the offending. ^(b)^ Refers to cases in which a mental illness was suspected, whether due to a post-offending in-person assessment or, in cases where the offender died during the offending, an assessment of the offender’s personal history, writings etc.

## Discussion

This review drew upon published case studies to compare the characteristics and motivations of individuals who killed family members and the perpetrators of grievance-fuelled homicides, and to examine the extent to which these findings supported the inclusion of family homicides under the broader umbrella of lone-actor grievance-fuelled homicide. This study found that a grievance or multiple grievances were present in over half the published case studies of fatal family violence, and these cases shared some notable similarities with individuals who committed grievance-fuelled non-familial homicides. This review supports the premise that a sub-group of fatal family violence offenders fall within the realm of lone actor grievance-fuelled violence more broadly, but there is another sub-group of FFV cases that are not motivated by grievance and have different offender, victim, and other characteristics. These results challenge existing assumptions that largely exclude family violence from LAGFH research on the basis of their relationship with the people they target (Lankford, 2012; Krouse and Richardson, 2015; Clemmow et al., 2020).

### Comparing LAGFH and FFV

#### Motivations

The observed differences in motivation between the grievance categories (whether family-related or otherwise) and non-grievance FFV was not unexpected. Grievance-fuelled violence is by definition violence motivated by a sense of injustice or being wronged.

LAGFH and grievance-fuelled FFV were differentiated only by the target of the perceived grievance (i.e., a family member rather than some other target), though grievances against family members were also seen in LAGFH cases with non-familial targets. A distinct group of homicides motivated by a desire for revenge and a sense of having been wronged accounted for 81.2% of the LAGFH cases and 66.7% of the GF-FFV cohort. Common amongst GF-FFV cases was the perception of threat and a need to defend themselves, which in turn was associated with grievance. In the grievance groups, such motivations typically arose from persecutory beliefs secondary to a psychotic illness. These motivations were not evident amongst non-grievance-fuelled FFV cases, whereas altruistic motivations were evident in 61.5% of cases. In this group there was less planning and violence appeared more often motivated by a belief that the victim could not survive without the suicidal offender.

The LAGFH construct rests on the premise that violence that occurs in different contexts can be thought about in a consistent manner because its share a common motivation and resultant emotional state: that is, resentment, outrage, blame and a desire for revenge. These shared characteristics are the foundation upon which those engaging in various kinds of lone actor killings have been conceptualised within the construct of LAGFV/H (Fox and Levin, 2003; McCauley et al., 2013; Meloy and Genzman, 2016; Taylor, 2018; Clemmow et al., 2020). These features were evident in the sub-group of GF-FFV, with blame and a desire for revenge being directed towards family members rather than an external target. James et al. (2022) have noted that rather than conflating motivations by focusing upon the target’s occupation (e.g., attacks on schools, workplaces or public figures), it has been more informative to consider the factors motivating the violent act. Such an approach has formed the basis for offender typologies and approaches to risk assessment in other forms of targeted violence such as stalking (Mackenzie et al., 2009) and sexual homicide. As Lussier and Cale (2014) noted in relation to sexual homicide, person-oriented, as opposed to variable-oriented approaches to understanding similarities and differences within a given population, are useful for clinical descriptions, intervention, and risk prediction. Notably, in their review of person-oriented approaches to understanding sexual murder, Higgs et al. (2017) also identified grievance as motivation in this specific context, and frequently grievance specifically involving resentment of women, suggesting a further potential area where grievance and homicide may intersect.

If motivation is central to the concept of lone actor grievance-fuelled violence then it seems logical to include fatal acts of grievance-fuelled violence that target family members within the construct of LAGFH. If cases are classified according to motivation (grievance-fuelled or not), this review suggests that approximately half of all familial homicides should be considered acts of grievance-fuelled violence.

#### Mental illness and stressors

There were similarities between the LAGFH and FFV groups with respect to diagnosed mental illness. Even where mental illness was not an immediate stressor, there was evidence that between half and three quarters of offenders across all three groups in this study had been diagnosed with mental disorders, and in a further 50% of those without a diagnosis at the time of the offence. There is an overrepresentation of mental illness amongst offenders in this study compared to the general population, consistent with the existing LAGFV/H research literature. This is also reflected in the literature on mental illness in a range of offenders, including lone actor terrorists and homicide offenders in general (Langman, 2009; Corner and Gill, 2015; Lankford, 2015a; Corner et al., 2016, 2018). The prevalence of mental health issues observed in the current study is also consistent with the literature on mental illness in those who kill family members. There is a higher rate of psychopathology in intimate partner homicide offenders relative to general population base rates (Belfrage and Rying, 2004), and rates of mental illness are higher again in family massacres (Oram et al., 2013; Kivisto, 2015).

This study suggests that the presence or absence of mental illness does not meaningfully distinguish cases of LAGFH and FFV. Mental illness may be present in both groups but plays a varying role (Peterson and Densley, 2020). Clearly, the presence of mental illness has not necessarily hindered the offender’s ability to form grievances or to plan and execute an attack (Gill et al., 2014). Indeed, in cases reviewed in this study some psychiatric symptoms can generate grievances, imagined or otherwise, and provide the motivation and commitment to attack. In other cases, mental illness appeared to play a less direct role, as a predisposing stressor that impacted on the offender’s resilience and coping strategies.

Stressors were evident in all cases in this study, regardless of motivation and victim type. In grievance-fuelled cases some stressors were directly related to the grievance (e.g., school shooting in the context of being bullied by peers) whilst others were cumulative factors in the offender’s social decline (e.g., loss of employment and financial hardship). Health problems, whether mental, physical or both, featured prominently in all homicide categories. There were active stressors at the time of the offence in approximately half of the FFV cohorts but were less than a third of LAGFH offenders. The presence of stressors in these cases is consistent with the broader LAGFH literature that suggests disappointments, frustrations and adverse experiences can propel individuals towards a grievance narrative (Rokach, 2017; Taylor, 2018; Clemmow et al., 2020). Stressors did not meaningfully distinguish between grievance-fuelled homicides in this study and family killings that were not motivated by grievance. This suggests that stress may simply be a precondition for homicides that are not motivated by instrumental gain and are not specific to grievance-fuelled homicide.

#### Offence behaviours

There were differences between the LAGFH and FFV cases in several offence behaviours. The LAGFH group was most likely to commit the attack in an indoor public location with the use of a weapon, often a firearm, and in almost two-thirds of cases resulted in the offender dying at the scene. The FFV groups, however, preferred the attack location of a private premises, and only used a weapon in three-quarters of the incidents. These weapons were also more varied than the LAGFH cases, utilising firearms as well as bladed instruments, blunt objects and other objects that may have been opportunistic, such as a phone cord. The FFV cases were also substantially less likely to culminate in the death of the offender than the LAGFH cases.

The observed differences in attack locations between the LAGFH and FFV cases aligns with existing literature and perhaps reflects opportunity and access to victims. Acts of FFV often occur in the home (Iratzoqui and McCutcheon, 2018) whereas the attack location in LAGFH cases is frequently a public setting, which may have some symbolic significance to the offender (Lankford, 2016; Böckler et al., 2018). The preference for firearms amongst LAGFH cases in this review was also not unexpected. These cases are often striving for higher fatality rates, making firearms an effective choice (see Lankford et al., 2019). A firearm can also be an appealing option for offenders with low skillsets in physical confrontations, or in cases with a power imbalance (victimisation by school bullies). Alternatively, in FFV where a physical power imbalance may already exist, offenders may prefer a more intimate weapon such as strangulation.

The use of a firearm also increases the likelihood of an offender committing suicide after the attack or being killed by police (Hagan et al., 2015). Suicidal motives can play a major role in offender behaviours (Lankford, 2015b). Almost two-thirds of LAGFH offenders in this study died at the scene, by suicide or police intervention. This is mirrored in LAGFH literature, where a similar outcome was noted in one-to two-thirds of LAGFH cases (Meloy et al., 2004; Lankford, 2015b; Taylor, 2018). Our review showed substantially lower rates of suicide for the FFV cases though this was dependent on the type of FFV. Intimate partner homicides perpetrated by males are highly correlated with suicide, with previous studies reporting suicide rates of approximately 50% in these cases (Buteau et al., 1993; Matias et al., 2020). Barnes (2000) suggested that homicide-suicides accounted for almost 90% of all lethal attacks in family contexts. Over half of these involved an intimate partner, and less commonly the offender’s children. Although homicide-suicide scenarios are prevalent in FFV. The differences noted between LAGFH and FFV offenders dying at the scene in this review may also have some relationship to the offence location. That is, killing within the home rather than in a more public setting might allow offenders time to consider their actions without external pressures or police interference.

Whilst some offence characteristics distinguished between LAGFH and FFV cases, LAGFH and GF-FFV were more similar to each other than to non-grievance FFV in their attack planning. Planning was evident in each of the LAGFH cases and almost all GF-FFV cases, though in only half of the non-grievance FFV cases. Acts of targeted violence in LAGFH research are generally accompanied by high levels of research and planning, though a precipitating crisis may be the catalyst for the final attack (Fox et al., 2011; Meloy and Pollard, 2017; Keatley et al., 2020; Capellan and Silva, 2021). These differences in attack planning between subsets of FFV have been described by others, from little to no planning (Dutton and Kerry, 1999; Stanton et al., 2000) to highly premeditated attacks (Johnson, 2006). Motivation (grievance) may be key to the observed discrepancies in premeditation in previous family violence literature.

#### Offender characteristics

There were generally more similarities than differences between groups in their demographic profile. Differences were apparent between grievance and non-grievance categories rather than between those who did or did not kill family members. Two variables of significance that separated the grievance from non-grievance categories in this study was the offender’s gender and the relationship of attack location to their country of birth. In both grievance-fuelled categories (LAGFH and GF-FFV) the majority of offenders were males, whereas over half of the non-grievance FFV cohort were females. Nearly 90% of grievance-fuelled attacks occurred in the offender’s country of birth, but fewer than two-thirds in the non-grievance FFV category.

The observed difference in country of birth between grievance and non-grievance FFV may reflect the case studies included in the non-grievance FFV category. Immigrants – especially women – may have experienced limited access to health and support services, diminished social networks and language and employment barriers (Sabri et al., 2018). When these stressors and potential risk factors are considered in conjunction with the profile of the non-grievance category of FFV (three-quarters female, highest rates of mental illness, especially mood disorders, and violence motivated by altruism towards their children), it suggests that these homicides are fundamentally different from those in the GF-FFV category.

Studies, supported by this review, have consistently found that most LAGFH offenders are male (Lankford, 2015b; Hamm and Spaaij, 2017; Liem et al., 2018; Capellan et al., 2019; Duwe, 2020; Kenyon et al., 2021). Similarly, most acts of homicides against family members are committed by males, though there is more gender discrepancy in parents who kill children (Daly and Wilson, 1988; Bourget et al., 2007; Liem and Koenraadt, 2008; Heide and Frei, 2010; Heide, 2013; Duwe, 2020). Previous research has found similar offending rates between males and females in family contexts, whilst others have reported a preponderance of males, typically involving non-biological relatives (Bourget and Gagné, 2005; Brookman and Nolan, 2006; Flynn et al., 2013). The inconsistencies may be explained by the conflation of motivations in previously studied FFV. The non-grievance-fuelled FFV cases in the current study found a higher proportion of female offenders. It is possible that the case study design resulted in an over-representation of maternal filicides, creating a bias in the reported female to male offender ratio (Bourget and Gagné, 2005). With the exception of one case, all female offenders in this study engaged in FFV, and each of these FFV incidents included the killing of children in the offender’s care.

### The presence of prior family violence amongst LAGFH offenders

To address the third research question, this study considered any evidence that those engaging in LAGFH had also engaged in family violence. Spencer and Stith (2018) found that prior family violence constitutes a risk factor for intimate partner homicide and that there is increasing anecdotal evidence of a history of family violence amongst mass killers (Everytown for Gun Safety, 2021; Freeman, 2017; Hamm and Spaaij, 2017; Taylor, 2018; Follman, 2019; McCulloch et al., 2019; Scaptura, 2019; DeVoe and Nicholson, 2020; McPhedran, 2020; Monckton Smith, 2020; Branigin, 2021; Rottweiler et al., 2021; Silva et al., 2021).

The current review considered any evidence that those engaging in LAGFH had also engaged in family violence. We found that this has not received much attention in the LAGFH literature to date, perhaps reflecting McCulloch et al.’ (2019) observation that violence in the family sphere tends to be discounted. However, the fact that prior family violence was poorly reported even in the FFV cases suggests that this may be a broader issue of inattention to the potential role of family violence as an antecedent to homicide.

### The defining features of grievance-fuelled fatal family violence

Twenty cases of FFV in this study were identified as “grievance-fuelled” based. Themes of revenge or having been wronged were prominent within the GF-FFV cases whereas other fatal family violence was more often motivated by altruism or reactionary frustration towards the victim. The key factor that differentiated motivations in GF-FFV and LAGFH cases was that the grievance-fuelled violence targeted a family member, rather than other targets or settings such as schools or minority groups.

In this review a quarter of the GF-FFV cases killed people other than family members. Each of these five cases involved fatal attacks on family members in the home before attacking a school or college. That most of these cases met the criteria for grievance-fuelled homicide (LAGFH or GF-FFV) is indicative of the overlap between two groups that have previously been considered conceptually distinct.

### Limitations

The current study relied on the availability of information gained from existing published cases. There was some selection bias in that the more unusual cases may have been chosen for publication (e.g., women who kill children versus men who kill partners). The database was also limited in its scope (English-language and liberal industrialised nations only). Such limitations are not unique to this study and were addressed by utilising a wide scope of samples, but specific screening criteria to ensure information accuracy. This review is limited also by the information included by the original authors and on their views and interpretation of motivations. The amount of missing data was problematic for several variables and hindered our ability to draw conclusions in some areas.

### Implications

This study supports the findings of research in other areas of targeted violence, in that rather than studying homicides according to the relationship between victim and offender, the victim’s sector of employment, location of the attack, or fatality count, it may be more useful to examine the factors motivating the attack, in particular grievance formation (James et al., 2022). The current study provides clear rationale for considering a sub-group of fatal family violence as a form of lone actor grievance-fuelled homicide, given the similarities in motivation between these two groups. The benefits of this approach are that the insights gained from research in one group may be transferable to others. Clemmow et al. (2020, p. 2) refer to “an overarching framework for guiding threat assessment,” and indeed evidence-based tools have been developed for lone actor grievance-fuelled violence that could inform the assessment of potential family homicide offenders and the development of preventative interventions.

## Data availability statement

The raw data supporting the conclusions of this article will be made available by the authors, without undue reservation.

## Author contributions

AC and TM contributed to the design of the study. AC organised and reviewed databases for initial sample selection. AC and MP reviewed cases for final inclusion. AC coded all data with some assistance from TM. AC wrote the first draft of the manuscript. All authors contributed to the manuscript revision, read, and approved the submitted version.

## Conflict of interest

The authors declare that the research was conducted in the absence of any commercial or financial relationships that could be construed as a potential conflict of interest.

## Publisher’s note

All claims expressed in this article are solely those of the authors and do not necessarily represent those of their affiliated organizations, or those of the publisher, the editors and the reviewers. Any product that may be evaluated in this article, or claim that may be made by its manufacturer, is not guaranteed or endorsed by the publisher.

## Footnotes

^1^Isolated dyads are pairs of individuals who "conceive, develop, and carry out activities without direct input from a wider network" (Gill et al., 2014, p. 426).

^2^Initial screening included German and Dutch language studies with the objective of having them translated by a multi-lingual colleague. However, this was not able to be conducted, so the review was confined to English language papers.
